# Supplementary material for: HIV envelope V3 region mimic embodies key features of a broadly neutralizing antibody lineage epitope
Source: Nat Commun. 2018 Mar 16;9:1111. doi: 10.1038/s41467-018-03565-6 (PMC5856820; doi:10.1038/s41467-018-03565-6)
Supplement: Supplementary file 1 — Supplementary Information(PDF 10001 kb) [file 41467_2018_3565_MOESM1_ESM.pdf]

Supplementary Information

for Fera et al.

HIV Envelope V3 Region Mimic Embodies Key Features of a Broadly Neutralizing Antibody Lineage Epitope

Supplementary Figure 1

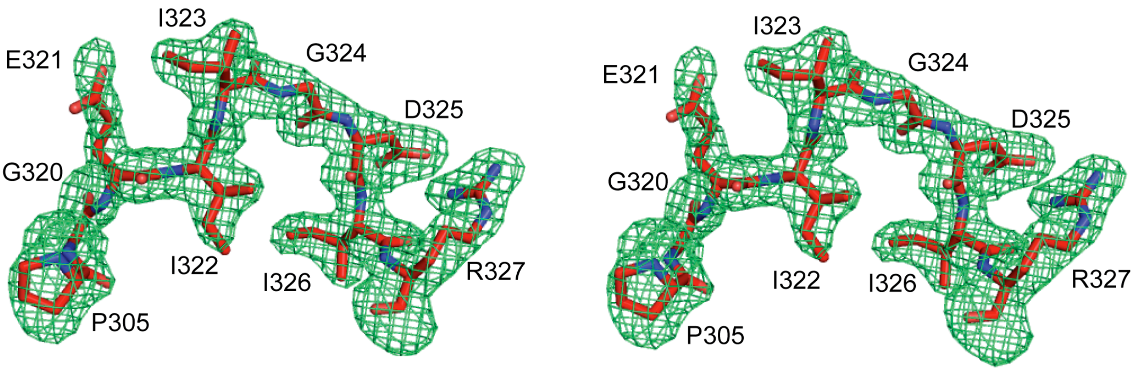

Figure S1. **Sample of electron density for Man$_9$-V3 that was determined in complex with DH270.6.** The volume, shown in stereoview, is from a 2Fo-Fc electron density map, contoured at 1.5 σ, in the region corresponding to the V3 glycopeptide residues 305, 320-327, which are continued at one end of the peptide.

Supplementary Figure 2

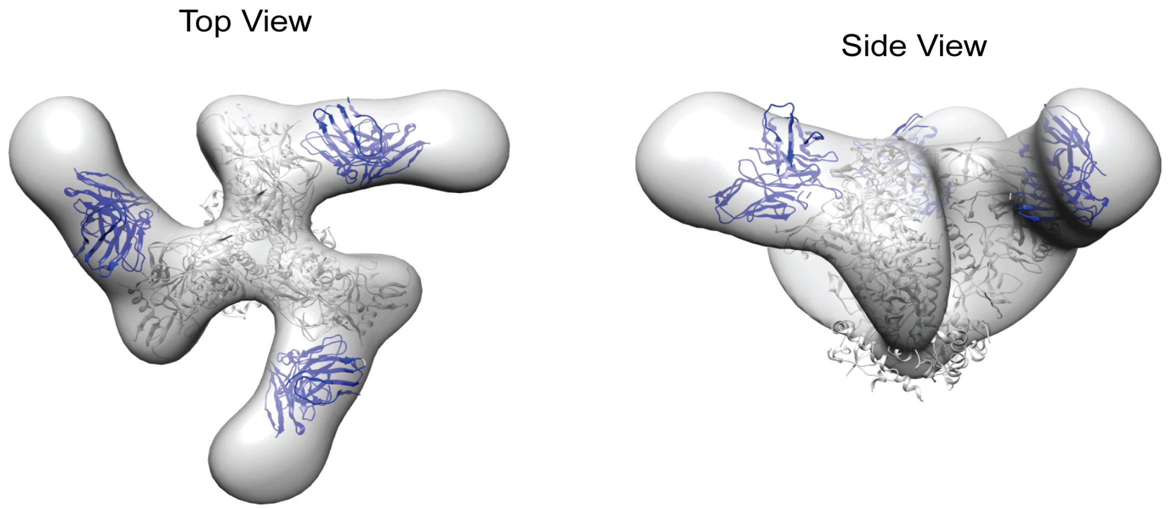

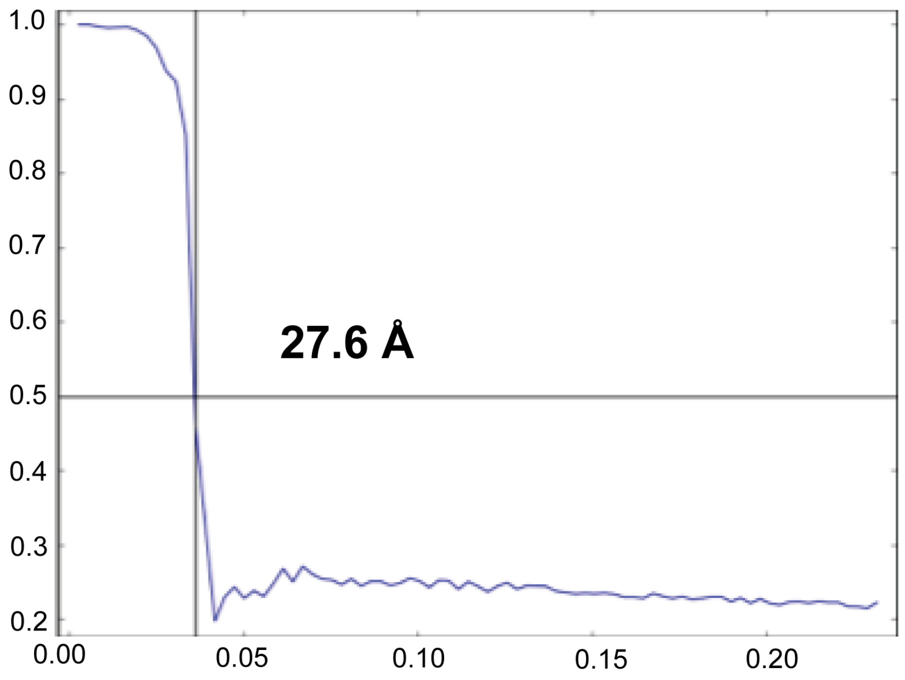

Figure S2. Negative Stain EM 3D Reconstruction of 92BR SOSIP.664 - DH270.6 Complex. Top and side views of the BG505 trimer (PDB ID: 5ACO) (gray) bound with DH270.6 scFv (blue), are shown. The FSC curve from which the resolution was obtained is shown.

Supplementary Figure 3

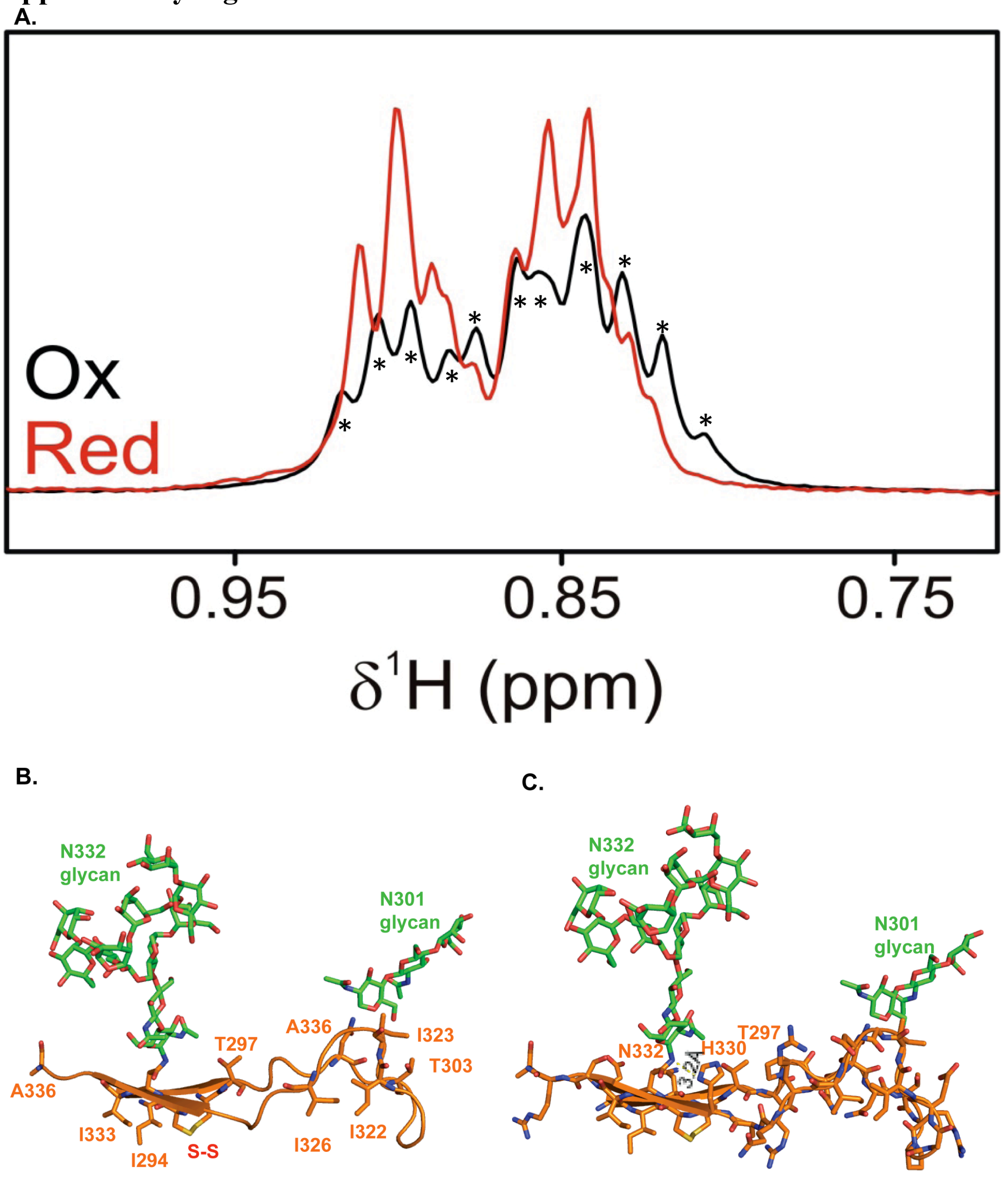

Figure S3. **Conformation of Man$_9$-V3.** (A) Methyl region of the 1D ^{1}H spectrum of the oxidized and reduced sample are shown in black and red, respectively and the peaks corresponding to the oxidized sample are highlighted with an asterisk (*). (B) The residues from Man$_9$-V3 (peptide is in orange and glycans are in green) that have methyl groups are shown as orange sticks and labeled. (C) The residues from Man$_9$-V3 (peptide is in orange and glycans are in green) that could be modified for increased stability are labeled.

Supplementary Figure 4

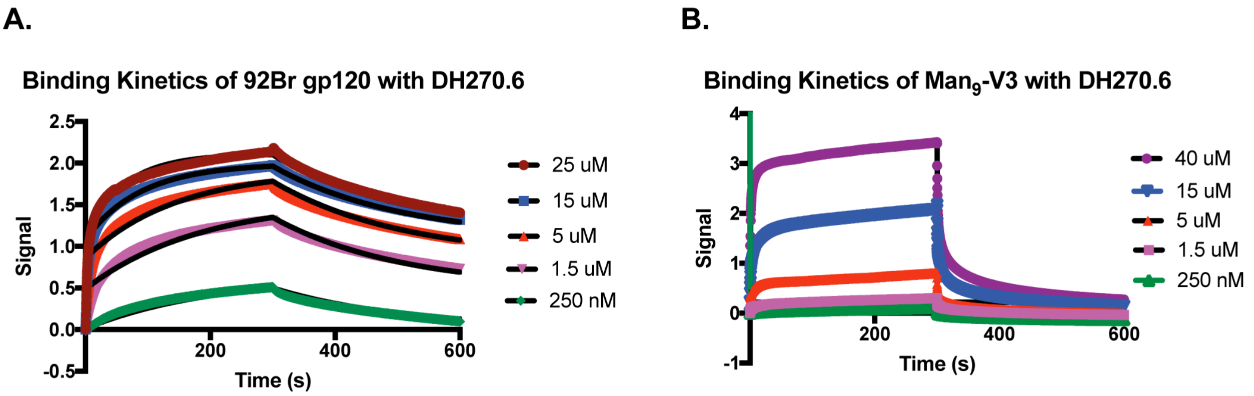

A: $K_D = 2. \pm 0.3$ μM
$k_a = 0.9 \pm 0.2 \times 10^4$ $M^{-1}s^{-1}$

B: $K_D = 7. \pm 0.4$ μM
$k_a = 1.0 \pm 0.05 \times 10^4$ $M^{-1}s^{-1}$

Figure S4. **DH270.6 Binding Kinetics with Man_9-V3 and HIV Env monomer**. Biolayer interferometry association curves are shown for (A) 0.2mg/mL biotinylated-Man_9-V3 tested with the indicated concentrations of DH270.6 Fab and (B) 0.2mg/mL DH270.6 Fab tested with the indicated concentrations of wild-type 92BR SOSIP.664. Errors are SEM, determined from duplicate measurements.

Supplementary Figure 5

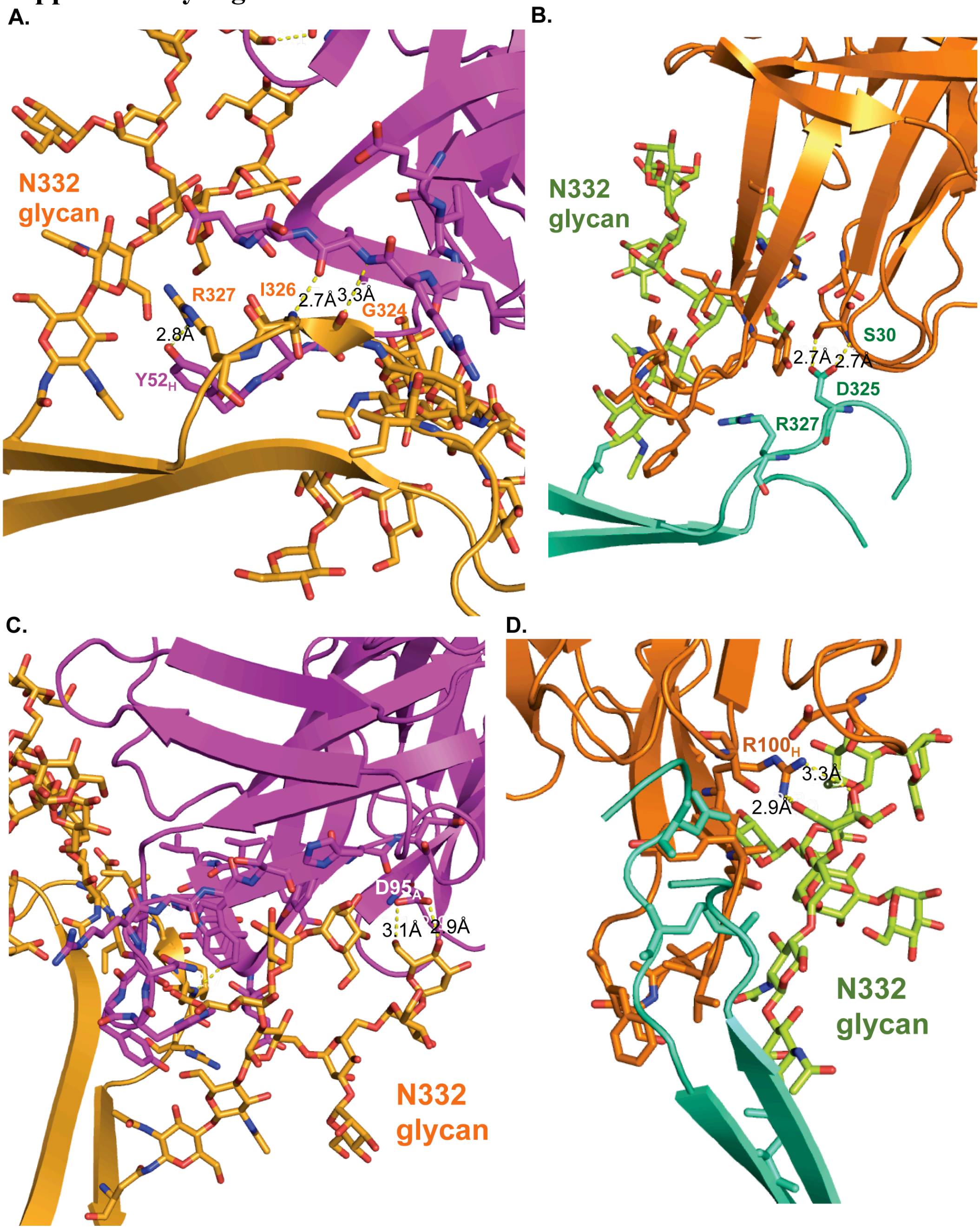

Figure S5. **Env Complexes with PGT124 and PGT128.** (A, C) PGT128 – BG505 SOSIP.664 complex (PDB ID: 5C7K) with only the V3 region (orange), N332 glycan (orange sticks) and PGT128 (magenta) are shown. (B, D) PGT124 – BG505 SOSIP.664 complex (PDB ID: 5T3S) with only the V3 region (green cyan), N332 glycan (light green sticks) and PGT124 (orange) are shown. Residues participating in hydrogen bonds are indicated.

Supplementary Figure 6

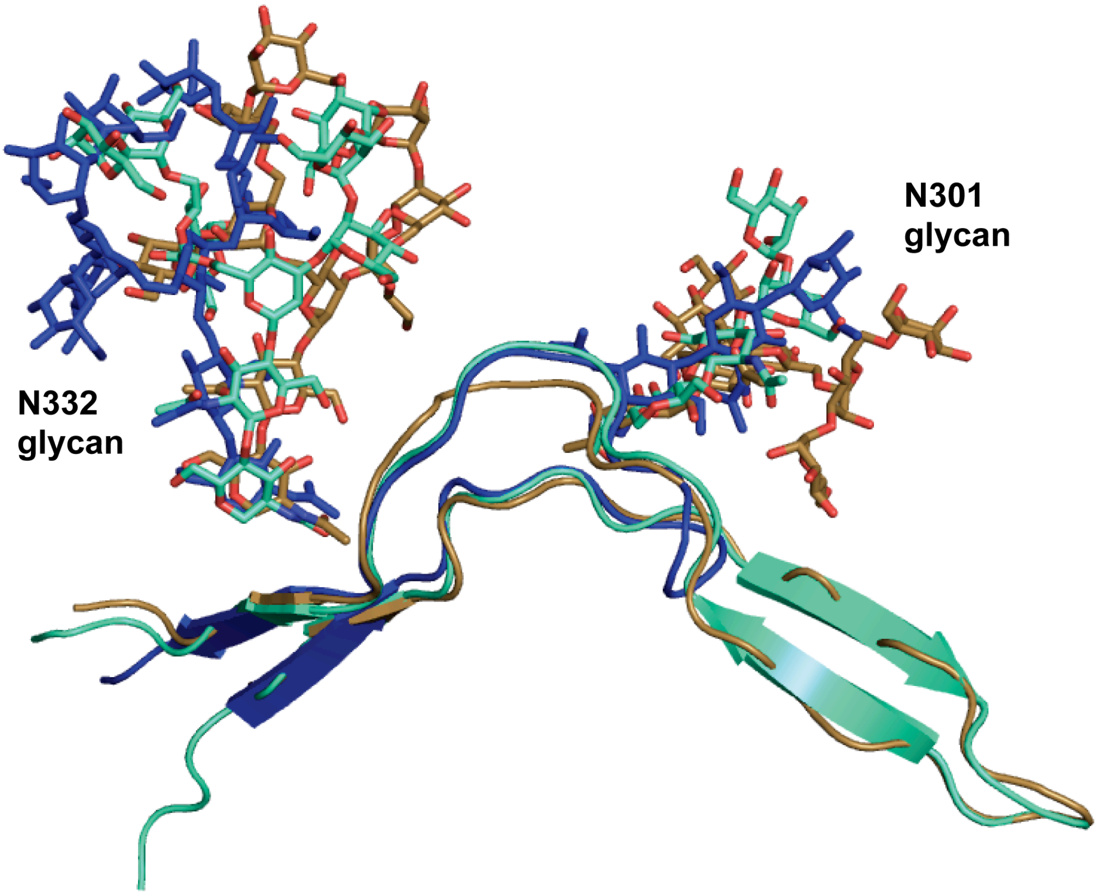

Figure S6. **N332 and N301 Glycan Conformations.** The V3 loops of the DH270.6 – Man_9-V3, PGT128 – BG505 SOSIP.664 (PDB ID: 5C7K), and PGT124 – BG505 SOSIP.664 MD39-10MUTA (PDB ID: 5T3S) are superposed. The N332 and N301 glycans are shown as sticks. Glycans and V3 loops from the complexes with DH270.6, PGT128, and PGT124 are shown in blue, brown, and light green, respectively.

Supplementary Figure 7

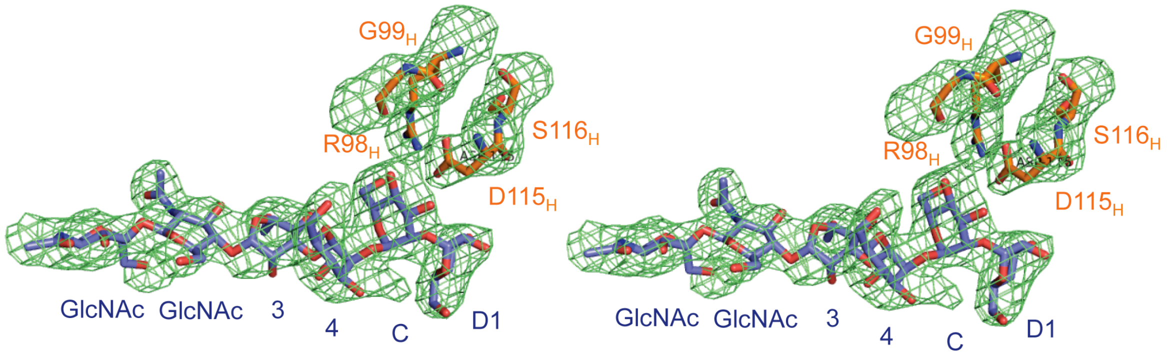

Figure S7. **Sample of electron density for the Man$_9$-DH270.3 complex.** The volume, shown in stereoview, is from a 2Fo-Fc electron density map, contoured at 1.5 σ, in the region corresponding to the D1 arm of Man$_9$ (blue) and residues 98-99 and 115-116 of DH270.3 (orange).

Supplementary Figure 8

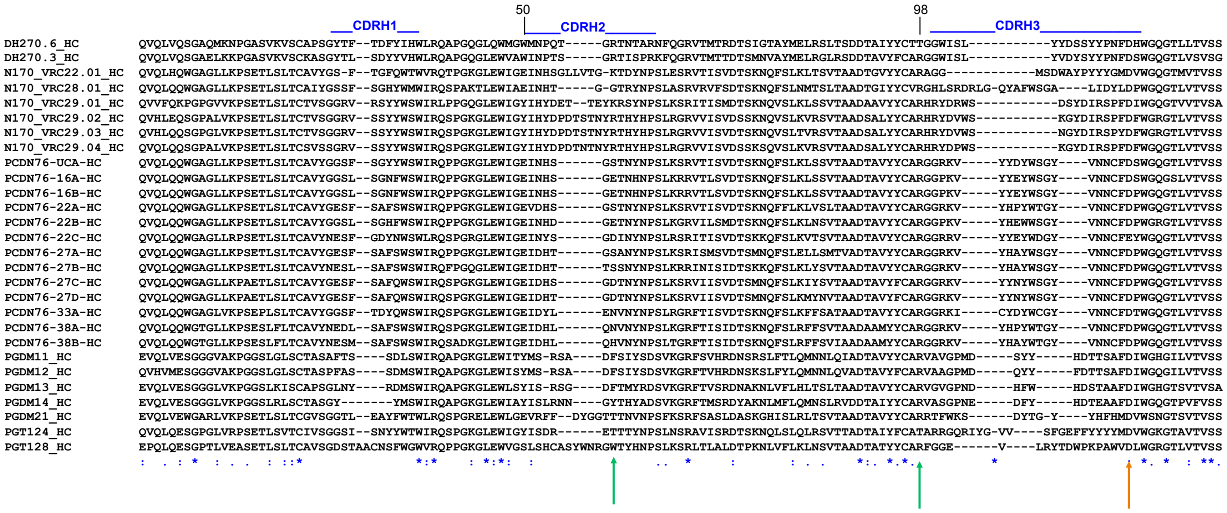

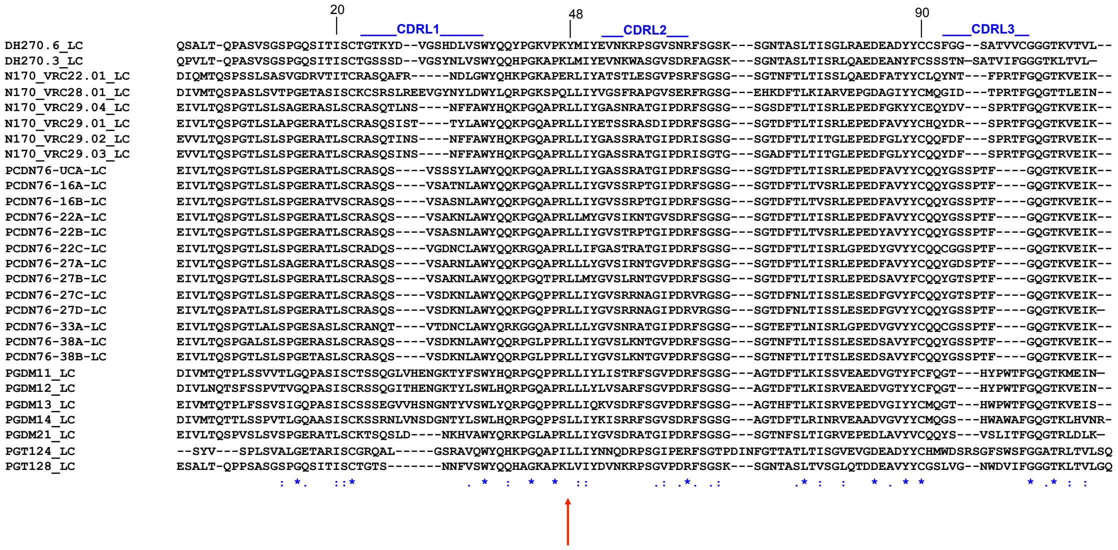

Figure S8. Sequence alignments of DH270 bnAbs with other N332-glycan dependent bnAbs. CDR loops are indicated. Critical improbable mutations discussed in the text are also shown with green and red arrows. The orange arrow indicates a conserved residue, important in DH270 lineage members for interacting with an improbable mutation.

Supplementary Table 1. Cα r.m.s.d.'s of Man_9-V3 Superposed on Intact HIV Env Trimers

PDB ID	Env	Fab(s) or scFv(s) Bound to Env	Cα r.m.s.d. (Å)
4ZMJ	BG505 SOSIP.664		0.99
5CEZ	BG505 SOSIP.664	PGT121	0.71
5I8H	BG505 SOSIP.664	PGT122	0.88
5FYK	JRFL SOSIP.664	PGT122, 35O22, VRC01	1.45
5FYJ	X1193.c1 SOSIP.664	PGT122, 35O22, VRC01	0.95
5T3S	BG505 SOSIP.664 MD39-10MUTA	PGT124	0.76
5ACO	BG505 SOSIP.664	PGT128	1.33
5V8L	BG505 SOSIP.664	PGT145, 3NBC117	0.81
5T3Z	BG505 SOSIP.664	IOMA, 10-1074	1.00

Supplementary Methods

General procedures for peptide and glycopeptide synthesis

Solid-phase peptide synthesis by Fmoc-strategy. Automated peptide synthesis was performed on an Applied Biosystems Pioneer continuous S3 flow peptide synthesizer. Peptides were synthesized under standard automated Fmoc protocols on Fmoc-Ala-TGT resin or TG Sieber resin. The deblock mixture was a mixture of 100:2:2 of *N,N*-Dimethylformamide/piperidine/DBU or a mixture of 80:20 of *N,N*-Dimethylformamide/piperidine with 0.1M OxymaPure. The following Fmoc amino acids from Novabiochem were employed: Fmoc-Ala-OH, Fmoc-Arg(Pbf)-OH, Fmoc-Asn(Dmcp)-OH, Fmoc-Asp(OPp)-OH, Fmoc-Asp(OtBu)-OH, Fmoc-Asp(OAll)-OH, Boc-Cys(Trt)-OH, Fmoc-Cys(Trt)-OH, Fmoc-Gln(Dmcp)-OH, Fmoc-Glu(OtBu)-OH, Fmoc-Gly-OH, Fmoc-His(Trt)-OH, Fmoc-Ile-OH, Fmoc-Leu-OH, Fmoc-Lys(Boc)-OH, Fmoc-Lys(Biotin)-OH, Fmoc-Phe-OH, Fmoc-Pro-OH, Fmoc-Ser(tBu)-OH, Fmoc-Thr(tBu)-OH, Fmoc-Tyr(tBu)-OH, Fmoc-Val-OH. The following pseudoproline dipeptide from Novabiochem was used: Fmoc-Ile-Ser($\Psi^{Me,Me}$ pro)-OH.

Acid-labile protecting group removal

Cocktail R. Peptides were subjected to Cocktail R consisting of trifluoroacetic acid (90% by volume), thioanisole (5% by volume), 1,2-ethanedithiol (3% by weight), and anisole (2% by volume) for 90 min. The resulting solution was poured in ice-cold diethyl ether to give a white precipitate, which was centrifuged. The precipitate was resuspended in ice-cold diethyl ether and centrifuged again twice. The supernatant was discarded and the precipitate was solubilized in water/acetonitrile (1:1, 0.05% trifluoroacetic acid), lyophilized and the resulting solid was purified by RP-HPLC.

RP-HPLC

All separations involved a mobile phase eluent of 0.05% TFA (v/v) in water (solvent A) / 0.04% TFA in acetonitrile (solvent B).

UPLC LC-MS analytical separations were performed using a Waters Acquity system equipped with an Acquity UPLC BEH C4 column (100 x 2.1 mm) or BEH C8 column (100 x 2.1 mm).

Preparatory RP-HPLC separations were performed using a WATERS 2545 Binary Gradient Module equipped with a WATERS 2996 Photodiode Array Detector using Waters C8 X-Bridge column (150 x 19 mm) at a flow rate of 16 mL/min.

Native chemical ligation (NCL) buffer

The buffer required for native chemical ligation (NCL) was freshly prepared prior to the reaction. Na_2HPO_4 (56.6 mg, 0.4 mmol) was solubilized in water (1 mL), Guanidine·HCl (1.146 g, 12 mmol), and TCEP·HCl (10.8 mg, 0.04 mmol) were then added and solubilized. The pH was adjusted to 7 with a solution of NaOH (5 M, 20 µL). After 15 min degassing with argon, 4-mercaptophenylacetic acid (MPAA) (67 mg, 0.4 mmol) was added and the pH was adjusted to 7.2 with a solution of NaOH (5 M, 120 µL). After 15 min degassing the solution was ready for use.

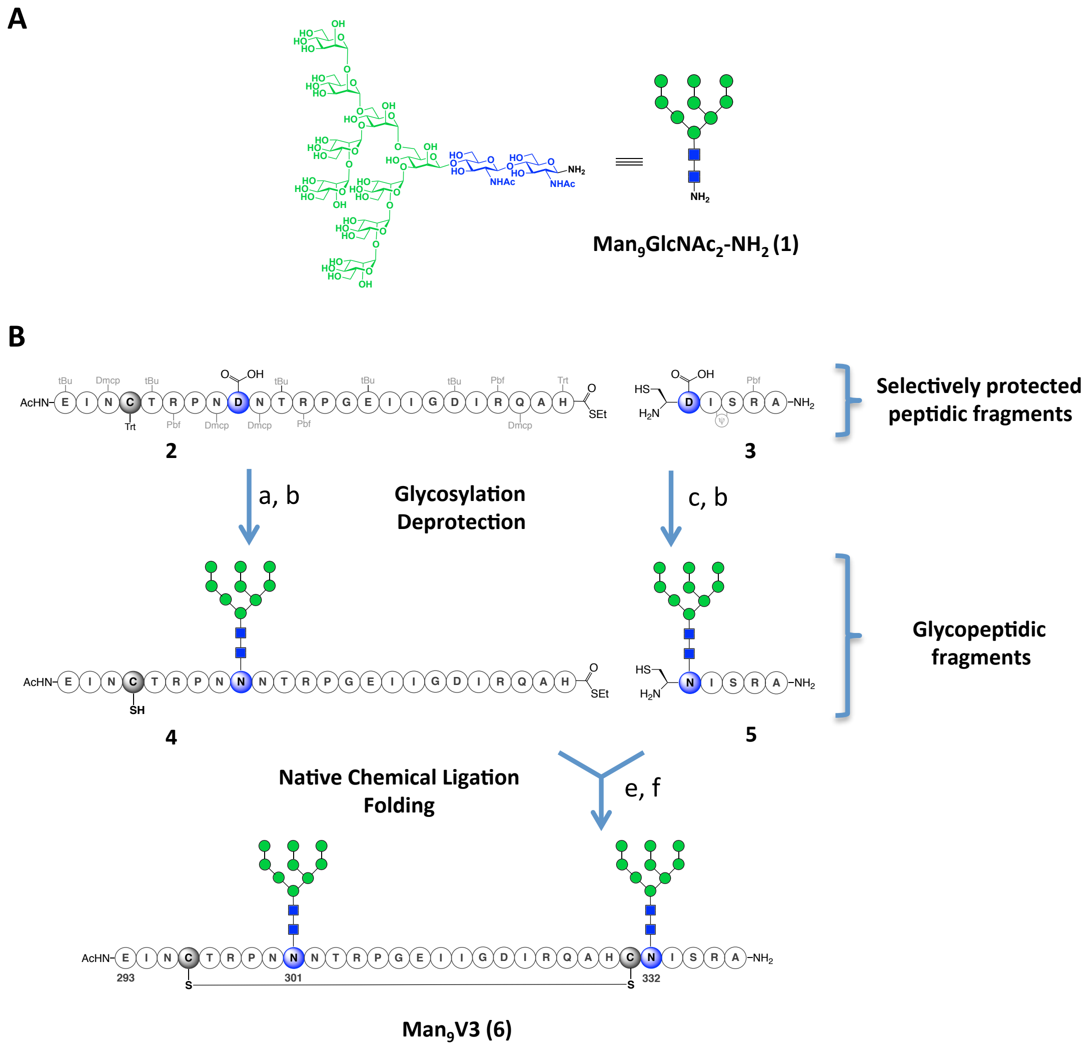

Scheme S1. (**A**) Chemical structure of Man$_9$GlcNAc$_2$–NH$_2$ (**1**). (**B**) Synthesis of Man$_9$V3 Glycopeptide **6** – reagents and conditions: (a) Man$_9$GlcNAc$_2$–NH$_2$ (**1**), PyAOP, DIEA, DMSO; (b) Cocktail R, 36% (2 steps); (c) Man$_9$GlcNAc$_2$–NH$_2$ (**1**), PyAOP, DIEA, DMSO; (d) Cocktail R, 49% (2 steps); (e) 6 M Gnd·HCl, 200 mM Na_2HPO_4, 200 mM MPAA, 20 mM TCEP·HCl, pH 7.2; (e) 0.1 M Gnd·HCl, pH 7, 68% (2 steps).

Man$_9$GlcNAc$_2$-NH$_2$ (1)

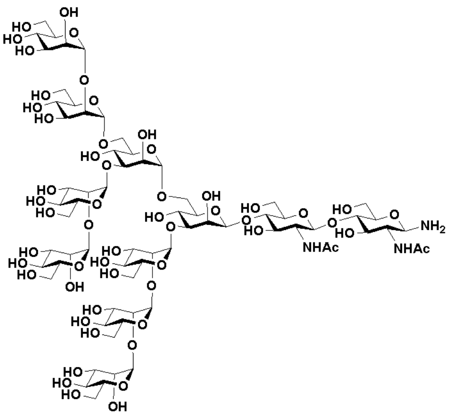

$Man_9GlcNAc_2$-OH was dissolved in water (5 mL) and added to $(NH_4)HCO_3$ (6 g, BioUltra, 99.5% (T), Cat. No. 09830 Fluka). The resultant slurry was warmed to 40 °C and stirred very slowly at this temperature for three days. After three days, the clear supernatant was filtered through a plug of cotton. The remaining material was rinsed with the same amount of cold water (2 x 5 mL), filtered, pooled with the clear supernatant, immediately frozen and lyophilized. The lyophilization was deemed complete when the mass of the product remained constant. This provided the glycosyl amine **1** as a white solid (quantitative). $Man_9GlcNAc_2$-NH_2 was stored at room temperature on the lyophilizer.

Protected *N*-terminal fragment (2)

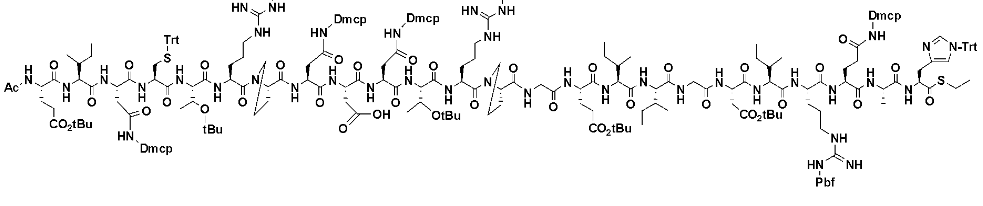

Upon completion of automated synthesis on 0.1 mmol of Fmoc-Ala-NovaSynTGT resin, the peptide-resin was subjected to acetylation. The peptide-resin was washed with *N,N*-

Dimethylformamide into a peptide synthesis vessel and treated with acetic anhydride (183 μL, 2 mmol), *N,N*-Diisopropylethylamine (384 μL, 2.2 mmol) in *N,N*-Dimethylformamide (2 mL) for 25 min. The peptide-resin was then washed with *N,N*-Dimethylformamide and methanol.

After drying, the resin was subjected to a cleavage cocktail (3:7 of hexafluoroisopropanol/methylene chloride) 3 times for 30 min. The resulting portions of cleavage solution were pooled and concentrated at room temperature. The oily residue was precipitated with water and the resulting mixture was immediately lyophilized to afford the peptide as a white solid (365 mg, 84% yield).

-Thioester incorporation. To a solution of this peptide (365 mg, 83.8 μmol) in chloroform (7.3 mL) was added EDC (37.3 μL, 212.5 μmol), HOOBt (35 mg, 213.3 μmol) and finally H-His(Trt)-SEt·HCl (**8**) (120 mg, 251 μmol). The mixture was stirred for 1 h 30 min at room temperature and precipitated with cold diethyl ether (90 mL). The precipitate was centrifugated and the pellet dried on high vacuum.

-Asp(OPp) selective deprotection. The resulting peptide was solubilized in methylene chloride (15 mL) at 0°C and methylene chloride/trifluoroacetic acid (4:1, 1.6 mL) was added dropwise. The reaction was stirred for 45 min at 0 °C and precipitated with cold diethyl ether (180 mL). After centrifugation, the resulting product was purified by size-exclusion chromatography (Sephadex LH-20, 1:9, methanol/methylene chloride). The product containing fractions were pooled and concentrated, resuspended in water/acetonitrile (1:1, 0.05% trifluoroacetic acid), and freeze-dried. Lyophilization provided peptide **2** (234 mg, 60% yield over two steps) as a white solid.

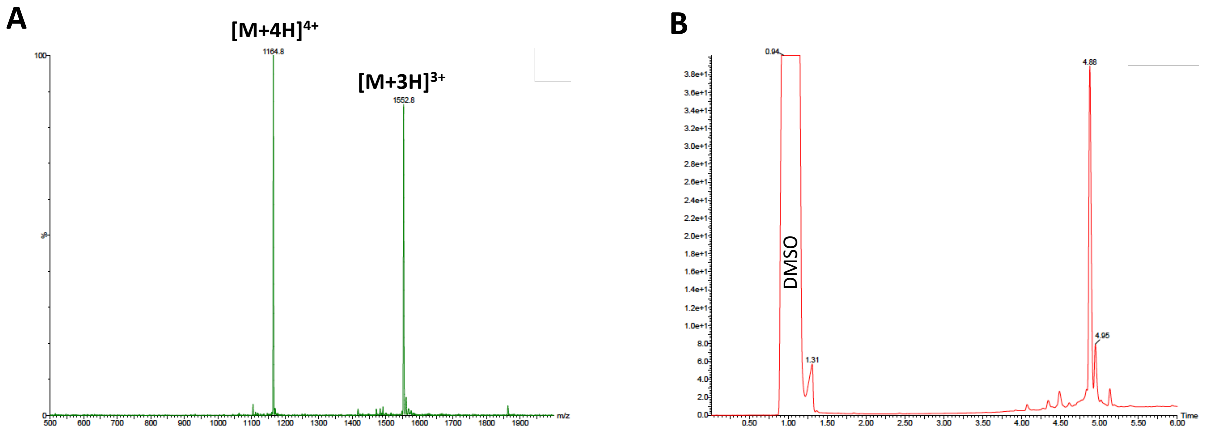

A - ESI-MS of compound **2**. ESI calculated for $C_{236}H_{345}N_{39}O_{48}S_5$ $[M+3H]^{3+}$ m/z: 1553.27, found: 1552.64. $[M+4H]^{4+}$ m/z: 1165.2, found: 1164.68; **B** - UV trace from UPLC analysis of compound **2**; gradient: 50% to 99% acetonitrile/water over 5 min at a flow rate of 0.3 mL/min, BEH C4 column.

Protected *C*-terminal fragment (3)

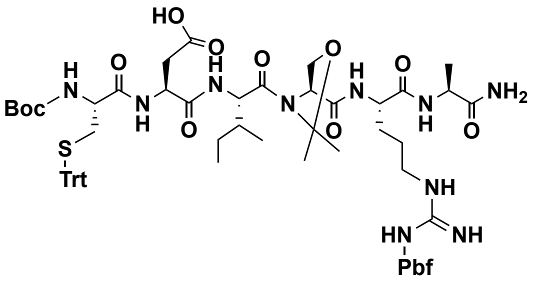

Upon completion of automated synthesis on 0.2 mmol of TG Sieber resin, the peptide-resin was subjected to deallylation. The peptide-resin was washed with a mixture of methylene chloride/*N,N*-Dimethylformamide (1:1) into a peptide synthesis vessel and treated with

Pd(PPh$_3$)$_4$ (10 mg, 8.6 µmol) and phenylsilane (100 µL, 0.8 mmol) in methylene chloride/*N,N*-Dimethylformamide (1:1, 5 mL). After 20 min, the Pd(PPh$_3$)$_4$/phenylsilane treatment was repeated twice. The peptide-resin was then washed with methylene chloride/*N,N*-Dimethylformamide and methanol. After drying, the peptide-resin was subjected to a cleavage cocktail (3:97 of trifluoracetic acid/methylene chloride, 4 mL) 5 times for 5 min. The resulting portions of cleavage solution were systematically pooled in cold diethyl ether and concentrated. The resulting product was purified by size-exclusion chromatography (Sephadex LH-20, 15:85, methanol/methylene chloride). The product containing fractions were pooled and concentrated, resuspended in a minimum amount of trifluoroethanol, precipitated with water and finally freeze-dried. Lyophilization provided peptide **3** (110 mg, 43% yield) as a white solid.

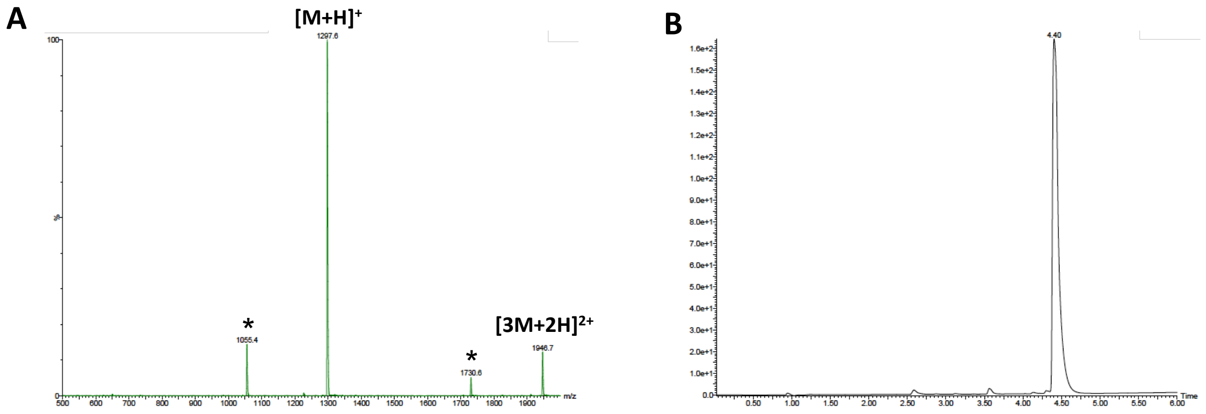

A - ESI-MS of compound **3**. ESI calculated for $C_{65}H_{88}N_{10}O_{14}S_2$ $[M+H]^+$ m/z: 1297.6, found: 1297.6; $[3M+2H]^{2+}$ m/z: 1946.8, found: 1946.7. The star (∗) indicates the product missing the cysteine trityl group which is lost during the ionisation process. **B** - UV trace from UPLC analysis of compound **3**; gradient: 20% to 90% acetonitrile/water over 5 min at a flow rate of 0.3 mL/min, BEH C4 column.

Man$_9$GlcNAc$_2$ *N*-terminal fragment (4)

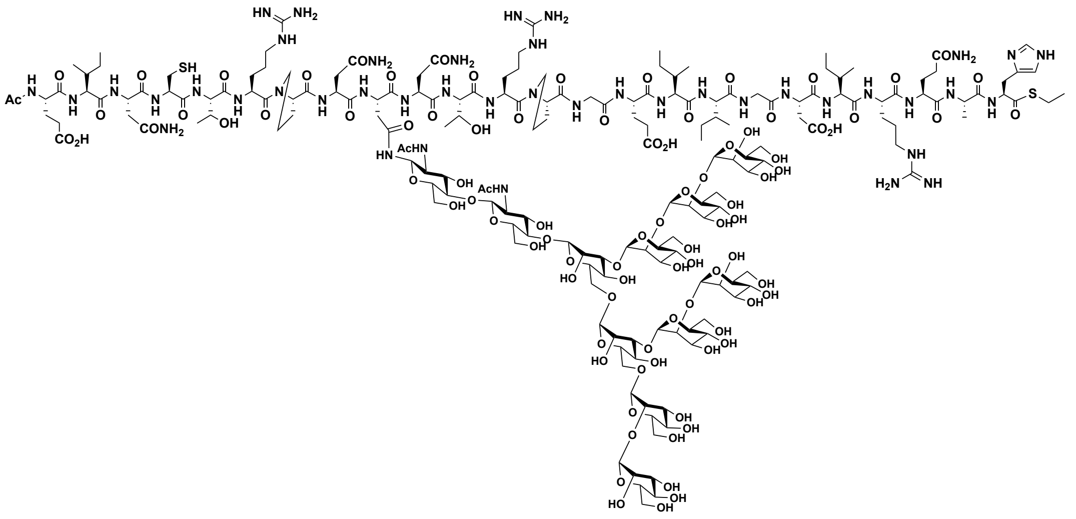

Peptide **2** (60 mg, 13.32 µmol, 1.3 equiv) and glycosyl amine **1** (19.36 mg, 10.28 µmol, 1 equiv) were combined and solubilized in anhydrous dimethyl sulfoxide (260 µL) and *N*,*N*-Diisopropylethylamine (23.2 µL, 133.2 µmol, 10 equiv). To this mixture, a freshly prepared solution of PyAOP in anhydrous dimethyl sulfoxide (37.68 mg, 72 µmol, 5.4 equiv, in 120 µL) was added. The solution turned a deep, golden-yellow color and this was stirred for 30 min. The reaction mixture was then frozen and lyophilized.

The glycopeptide was then subjected to Cocktail R (4 mL) for 1 h 15 min. The peptide was precipitated, centrifugated, resuspended and lyophilized as described in the general procedure. The resulting solid was purified to homogeneity by RP-HPLC (C8 X-bridge semiprep, 20% to 40% acetonitrile/water over 30 min, 16 mL/min). Product eluted at 13.27 min. Lyophilization of the collected fractions provided peptide **4** (17.4 mg, 36% yield over two steps) as a white solid.

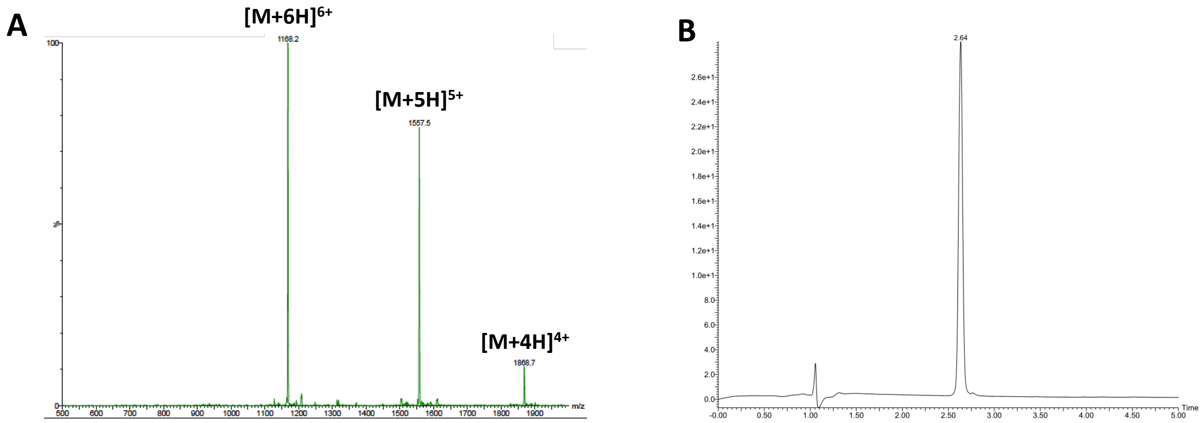

A - ESI-MS of compound **4**. ESI calculated for $C_{185}H_{306}N_{42}O_{93}S_2$ $[M+4H]^{4+}$ m/z: 1868.3, found: 1869.3; $[M+5H]^{5+}$ m/z: 1557.92, found: 1557.59; $[M+6H]^{6+}$ m/z: 1168.69, found: 1168.13; **B** - UV trace from UPLC analysis of compound **4**; gradient: 10% to 60% acetonitrile/water over 5 min at a flow rate of 0.3 mL/min, BEH C8 column.

Man$_9$GlcNAc$_2$ *C*-terminal fragment (5)

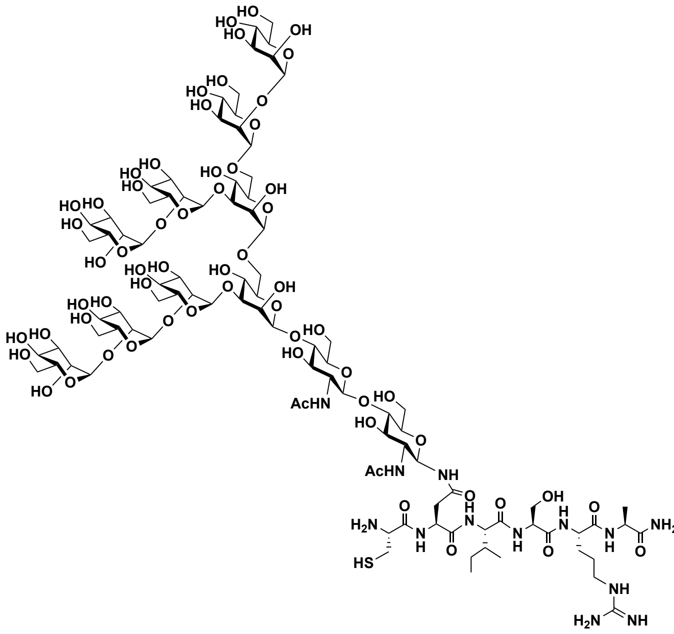

Peptide **3** (17.8 mg, 13.72 µmol, 1.45 equiv) and glycosyl amine **1** (17.8 mg, 9.45 µmol, 1 equiv) were combined and solubilized in anhydrous dimethyl sulfoxide (210 µL) and *N*,*N*-Diisopropylethylamine (21.4 µL, 123 µmol, 13 equiv). To this mixture, a freshly prepared solution of PyAOP in anhydrous dimethyl sulfoxide (34.5 mg, 66.2 µmol, 7 equiv, in 105 µL) was added. The solution turned a deep, golden-yellow color and this was stirred for 30 min. The reaction mixture was then frozen and lyophilized. The glycopeptide was then subjected to Cocktail R (1 mL) for 1 h 30 min. The peptide was precipitated, centrifugated, resuspended and lyophilized as described in the general procedure. The resulting solid was purified to homogeneity by RP-HPLC (C8 X-bridge semiprep, 5% to 40% acetonitrile/water over 30 min, 16 mL/min). Product eluted at 12.38 min. Lyophilization of the collected fractions provided peptide **5** (11.8 mg, 49% yield over two steps) as a white solid.

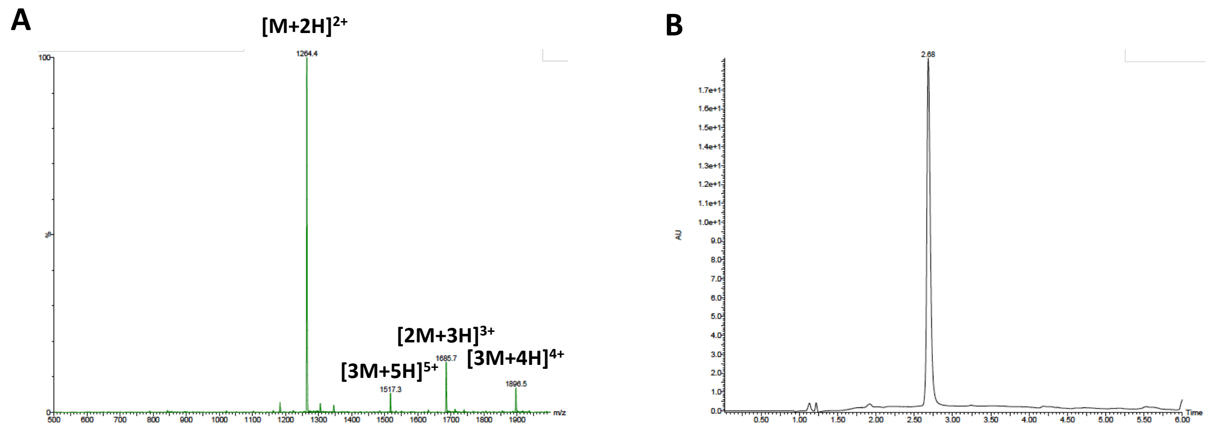

A - ESI-MS of compound **5**. ESI calculated for $C_{95}H_{163}N_{13}O_{63}S$ $[M+2H]^{2+}$ m/z: 1264.72, found: 1264.4; $[3M+5H]^{5+}$ m/z:1516.5, found: 1517.3; $[2M+3H]^{3+}$ m/z: 1685.0, found: 1685.7; $[3M+4H]^{4+}$ m/z:1895.6, found: 1896.5. **B** - UV trace from UPLC analysis of compound **5**; gradient: 1% to 30% acetonitrile/water over 5 min at a flow rate of 0.3 mL/min, BEH C8 column.

Man$_9$V3 (6)

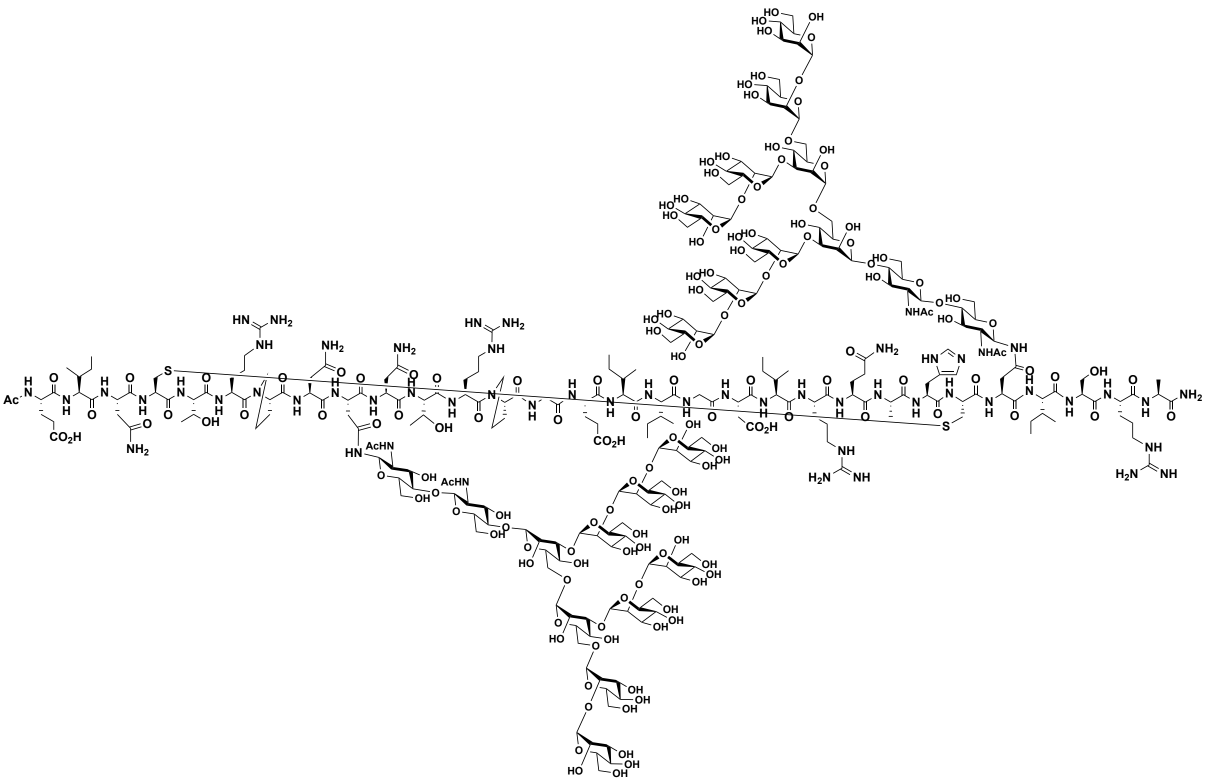

Freshly purified *N*-terminal fragment **4** (63 mg, 13.48 μmol) and *C*-terminal fragment **5** (47 mg, 18.59 μmol) were combined and solubilized in NCL buffer (1.73 mL, prepared as described in general procedure). To this mixture was added neutral TCEP solution (0.5 M, 192 μL). After 19 h the mixture was diluted dropwise with acetonitrile/water (1:1, 0.05% trifluoroacetic acid) and desalted by size exclusion chromatography (Bio-Gel P-4, Medium, acetonitrile/water (1:4, 0.05% trifluoroacetic acid)). After lyophilization the peptide was submited to air oxidation to form the disulfide bridge. The crude peptide was solubilized in a guanidine buffer at neutral pH (0.1 M, 30 mL). After 12h the reaction was quenched with 2 drops of trifluoroacetic acid and purified to homogeneity by RP-HPLC (C8 X-bridge semiprep, 5% to 40% acetonitrile/water over 30 min, 16 mL/min). Product eluted at 18.33 min. Lyophilization of the collected fractions provided **6** (65 mg, 68% yield over two steps)

as a white solid.

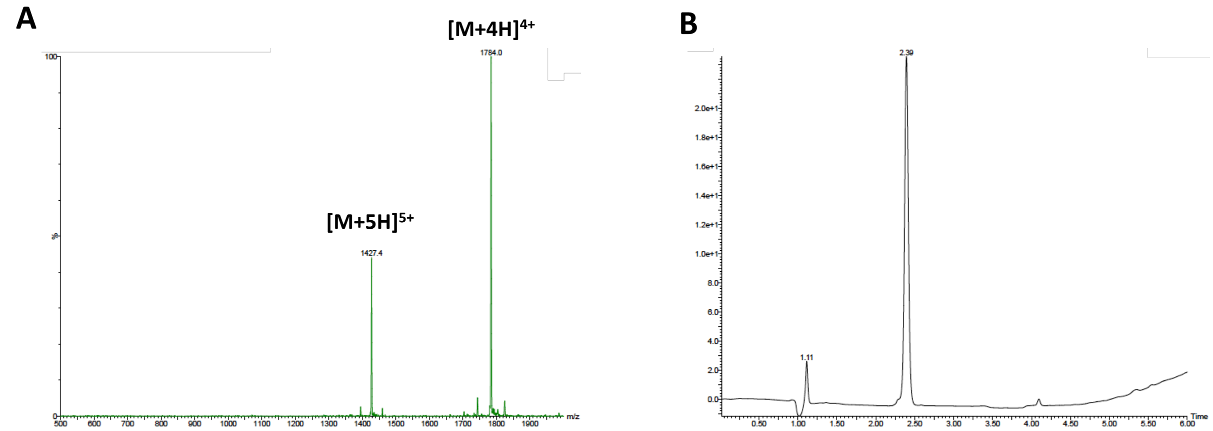

A - ESI-MS of compound **6**. ESI calculated for $C_{278}H_{461}N_{55}O_{156}S_2$ $[M+4H]^{4+}$ m/z: 1427.8, found: 1427.4; $[M+5H]^{5+}$ m/z: 1784.5, found: 1784.0. **B** - UV trace from UPLC analysis of compound **6**; gradient: 10% to 60% acetonitrile/water over 5 min at a flow rate of 0.3 mL/min, BEH C8 column.

H-His(Trt)-SEt·HCl (8)

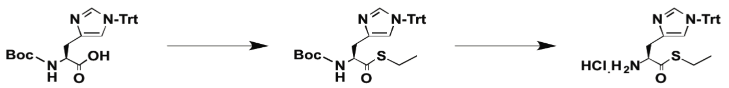

7 8

Boc-His(Trt)-OH (2.69 g, 5.4 mmol) and HOBt (2.2 g, 14.4 mmol) were solubilized in methylene chloride (27 mL). To this solution EDC (972 µL, 5.5 mmol) was added and the mixture was stirred for 15 min before addition of ethanethiol (1.96 mL, 27.2 mmol). The reaction mixture was stirred for 19h, concentrated in vacuo and purified by flash chromatography (0-15% methanol/methylene chloride) to afford after concentration Boc-

His(Trt)-SEt (**7**) (1.055 g, 1.95 mmol, 36% yield) as a white solid.

Boc-His(Trt)-SEt (588 mg, 1.09 mmol) was directly solubilized in a solution of HCl in dioxane (4 M, 12 mL) at 0 °C. After 3 h at 0 °C, the solution was concentrated with a stream of argon and lyophilized to afford H-His(Trt)-SEt·HCl (**8**) as a white solid (518 mg, quantitative yield).

^{1}H NMR (600 MHz, dimethyl sulfoxide -d_6) δ 8.72 (s, 1H), 7.54 – 7.39 (m, 9H), 7.35 (s, 1H), 7.28 – 7.10 (m, 6H), 4.62 (t, J = 7.3 Hz, 1H), 3.22 (d, J = 7.3 Hz, 2H), 2.89 (q, J = 7.3 Hz, 2H), 1.14 (t, J = 7.4 Hz, 3H).

^{13}C NMR (150 MHz, dimethyl sulfoxide -d_6) δ 195.4, 140.7, 137.3, 129.3, 128.7, 122, 77.2, 72.2, 70.6, 66.4, 60.2, 57.3, 43.7, 34.2, 27.1, 23.4, 14.4.
